# Evaluation of drug mechanism and efficacy of a novel anti-angiogenic agent, TTAC-0001, using multi-modality bioimaging in a mouse breast cancer orthotopic model

**DOI:** 10.1371/journal.pone.0187063

**Published:** 2018-01-25

**Authors:** Jinil Kim, Sang Hyun Choi, Su Jung Ham, Young Chul Cho, Seul-I Lee, Jeeheon Kang, Dong-Cheol Woo, Weon Sub Lee, Jin-San Yoo, Kyung Won Kim, Yoonseok Choi

**Affiliations:** 1 Department of Radiology and the Research Institute of Radiology, University of Ulsan College of Medicine, Asan Medical Center, Seoul, Korea; 2 Bioimaging Center, Asan Institute for Life Sciences, Asan Medical Center, Seoul, Korea; 3 PharmAbcine; Inc., Daejeon Bioventure Town, Daejeon Korea; 4 Medical Research Institute, Gangneung Asan Hospital, University of Ulsan College of Medicine, Gangwon-do, Korea; University of South Alabama Mitchell Cancer Institute, UNITED STATES

## Abstract

**Purpose:**

Targeting of vascular endothelial growth factor receptors (VEGFRs) has potential anti-angiogenic effects because VEGFR-2 is the major signaling regulator of VEGF/VEGFR pathways. We aimed to elucidate the drug mechanism and anti-tumor efficacy of TTAC-0001, a novel, fully human anti-VEGFR-2/KDR monoclonal antibody, in mouse orthotopic breast cancer model using multi-modal bioimaging.

**Materials and methods:**

We used orthotopic xenograft tumor model in which human breast cancer cells (MDA-MB-231) were injected into the right mammary fat pad of Balb/c nude mice. We investigated its biodistribution using serial fluorescence imaging after injecting fluorescent-labelled-drug and mode of action using Matrigel plug angiogenesis assays. The anti-tumor efficacy of drug was assessed using ultrasonography and bioluminescence imaging. Histopathologic analyses, including hematoxylin and eosin staining and immunohistochemistry with anti-CD31 and anti-Ki-67 antibodies, were performed. Each experiment had four groups: control, bevacizumab 10 mg/kg (BVZ-10 group), TTAC-0001 2 mg/kg (TTAC-2 group), and TTAC-0001 10 mg/kg (TTAC-10 group).

**Results:**

The TTAC-10 group showed good tumor targeting that lasted for at least 6 days and had a good anti-angiogenic effect with decreased hemoglobin content and fewer CD31-positive cells in the Matrigel plug. Compared with BVZ-10 and TTAC-2 groups, the TTAC-10 group showed the strongest anti-tumor efficacy, inhibiting tumor growth as detected by ultrasonography and bioluminescence imaging. The TTAC-10 group also showed the lowest viable tumor and micro-vessel areas and the lowest Ki-67 index in histopathologic analyses.

**Conclusion:**

We firstly demonstrated that TTAC-0001 effectively inhibited tumor growth and neovascularization in mouse orthotopic breast cancer model. It may provide a future treatment option for breast cancer.

## Introduction

Tumor angiogenesis is a potential target for anti-cancer therapy, as it plays an essential role in oxygen and nutrient supply [1, 2]. Antibodies against either vascular endothelial growth factors (VEGFs) or their receptors have been developed to target tumor angiogenesis [3, 4]. Bevacizumab, the first approved anti-angiogenic agent to target VEGF itself, achieved notable success as a novel targeted drug to treat several cancers, including colon, renal, and non-small cell lung cancer. Although its therapeutic efficacy is limited, it is generally used as part of a combination treatment regimen.

Targeting VEGF receptors (VEGFRs) is an alternative approach to inhibit angiogenesis in tumors. In particular, inhibition of the VEGFR-2/kinase insert domain receptor (KDR) has potential anti-angiogenic effects because VEGFR-2 is the major signaling regulator of VEGF/VEGFR pathways [5]. From this perspective, TTAC-0001, a human anti-VEGFR-2/KDR monoclonal antibody, was developed. TTAC-0001 binds to the VEGF-binding domain of VEGFR-2 and neutralizes the biological activity of VEGFR-2 by blocking the binding of VEGF [6]. Preclinical research revealed potential anti-tumor activity of TTAC-0001 in colorectal, non-small-cell lung cancer and glioblastoma tumor models [6–10]. Recently, a phase I clinical trial of TTAC-0001 was completed, and a phase IIa trial is ongoing. However, there have been no previous studies of TTAC-0001 for breast cancer.

In terms of chemotherapy for breast cancer, the major challenge is to develop an effective regimen for triple-negative breast cancer [11]. Bevacizumab had been incorporated as a second-line chemotherapy regimen for metastatic triple-negative breast cancer, but was revoked by the FDA due to inadequate therapeutic effect, suggesting that targeting the VEGF ligand itself may not be the best strategy [12, 13]. Therefore, an alternative approach, inhibition of VEGFR-2/KDR, is worthy of investigation for treatment of triple-negative breast cancer.

Bioimaging plays important roles in anti-cancer drug research [14, 15]. Multiple modalities such as magnetic resonance imaging, positron emission tomography have been applied for the oncologic drug development and each modality showed its own values to facilitate the development steps. Particularly, size measurement with ultrasonography (US) provides more accurate values of regressed tumor volumes [16, 17] when the drugs are treated and the utilization of optical imaging (fluorescence and bioluminescence) enables the studies of mode of action mechanisms and biodistribution of the drugs [18].

The validation of TTAC-0001 in triple-negative breast cancer has not been performed yet. Therefore, we aimed to investigate the drug mechanism and anti-tumor efficacy of TTAC-0001, a novel anti-angiogenic agent, in a mouse orthotopic breast cancer model using multi-modal bioimaging.

## Materials and methods

### Cells and drugs

Human breast cancer cells (MDA-MB-231) were purchased from the Korean Cell Line Bank (KCLB, Seoul, Korea) and were used in cell culture and animal experiments. For the establishment of MDA-MB-231+luc cells, MDA-MB-231 cells were transfected with a lentiviral vector containing the firefly luciferase reporter gene. Both MDA-MB-231 and MDA-MB-231+luc cells were cultured in Dulbecco’s modified Eagle’s medium (Welgene, Seoul, Korea) supplemented with 10% (v/v) heat-inactivated fetal bovine serum (GIBCO, Seoul, Korea).

TTAC-0001 (PharmAbcine, Daejeon, Korea; Table 1) was kindly provided by the manufacturer. Bevacizumab (Avastin®, Genentech, San Francisco, CA, USA) from clinically packaged vials was used for comparison of anti-angiogenic and anti-tumor efficacy.

**Table 1:** Drug summary.

Drug name	TTAC-0001
Phase	Phase IIa
Pharmacology description	TTAC-0001 is a fully human monoclonal antibody derived from a fully human single chain variable fragment phargelibrary. The TTAC-0001 neutralizes the vascular endothelial growth factor receptor2/vascular endothelial growth factor axis, which is a major mediator of tumor-angiogenesis, and therefore blocks angiogenesis and inhibits tumor growth and metastasis.
Route of administration	Intravenous
Chemical structure	Fully human IgG1 monoclonal antibody
Possible indication	Solid tumor (including glioblastoma, triple negative breast cancer)

### Animal experiments

**Fig 1:**
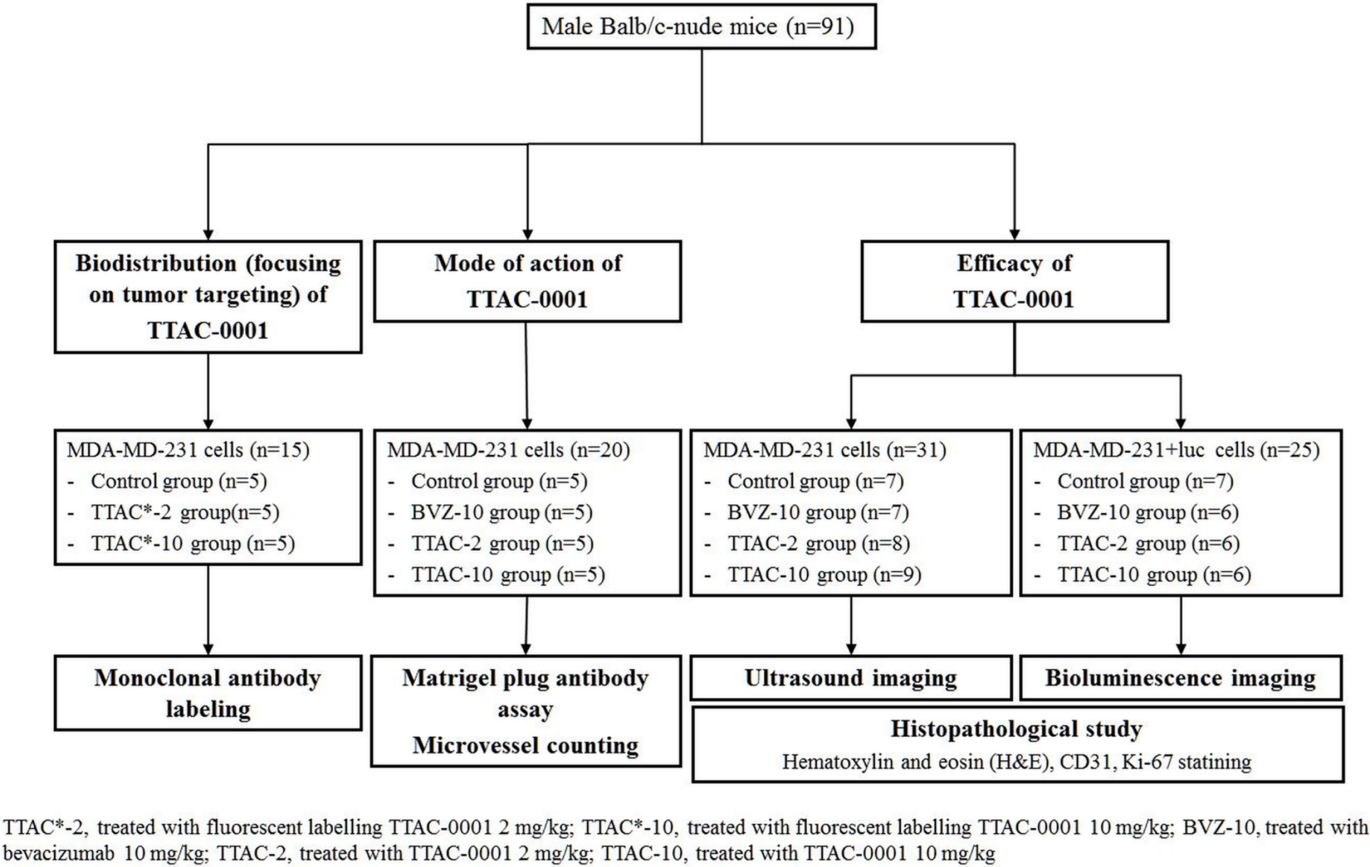
Flow chart of the experimental protocol of the animal study.

All animal experiments were performed according to our Institutional Animal Care and Use Committee approved protocol. The protocol was approved by the Committee on the Ethics of Animal Experiments of the Asan Medical Center (IACUC Number 2015-12-066). Female Balb/c nude mice (n = 91), 6 weeks old and weighing 20–25 g, were used. In our experiment, we used an orthotopic xenograft tumor model in which suspended cells were injected into the right mammary fat pad of the animals. The mice were treated with therapeutic agents approximately 2 weeks after tumor implantation, when the tumor diameter had reached approximately 5–6 mm in diameter (70–130 mm^3^ in volume). All therapeutic agents were dissolved in saline (0.1 mL) and injected intraperitoneally. Detailed information regarding the number of animals and experimental groups is presented in Fig 1.

Our study was composed of three experimental parts as follows:

1. Biodistribution of the drug, focusing on tumor targeting, by serial fluorescence imaging after injecting fluorescently labeled drug.
2. Use of Matrigel plug angiogenesis assays to evaluate the mode of action of the anti-angiogenic effect.
3. Monitoring of anti-tumor efficacy of the drugs by ultrasonography and bioluminescence imaging.

### Biodistribution for tumor targeting

#### Experimental groups

For the biodistribution assay, the animals were divided into three groups: a control group administered immunoglobulin G (n = 5), a TTAC*-2 group treated with fluorescently labeled TTAC-0001 2 mg/kg (n = 5), and a TTAC*-10 group treated with labeled TTAC-0001 10 mg/kg (n = 5).

#### Labeling of TTAC-0001

To perform fluorescent labeling of TTAC-0001, we used a commercial kit (Alexa Fluor® 647 Antibody Labelling Kit, Thermo Fisher Scientific, Waltham, MA, USA), according to the manufacturer’s protocol. Briefly, we induced a conjugation reaction between the Alexa Fluor® 647 reactive dye and TTAC-0001, and the purified labeled TTAC-0001 was collected. To determine the degree of labeling, the concentration of the dye was calculated based on absorbance at 280 and 650 nm. The collected labeled drug was stabilized at room temperature for 1 h and then intravenously injected into the mice.

#### Serial fluorescence imaging

Biodistribution of Alexa Fluor® 647-labeled TTAC-0001 was performed using the IVIS Lumina II machine (PerkinElmer, Waltham, MA, USA). Mice were anesthetized with 1% isoflurane (Forane®, Choongwae, Korea), and Alexa Fluor® 647-labeled TTAC-0001 was intravenously injected at doses of 2 and 10 mg/kg. Serial fluorescence images were then acquired at baseline and at 1, 2, 4, and 8 h and 1, 2, 4, and 6 days after drug injection. Imaging was performed with an exposure time of 1 s and an f/stop of 1. Using Living Image® 4.2 software (Caliper Life Sciences, Hopkinton, MA, USA), the peak total signal was measured by placing regions of interest (ROIs) at the tumors, which reflected the accumulated amount of Alexa Fluor® 647-labeled drug.

### Mode of action for the anti-angiogenic effect

#### Experimental groups

For evaluating the mode of action, the animals were divided into four groups that were treated with vehicle (control group, n = 5), bevacizumab 10 mg/kg (BVZ-10 group, n = 5), low-dose (2 mg/kg) TTAC-0001 (TTAC-2 group, n = 5), or high-dose (10 mg/kg) TTAC-0001 (TTAC-10 group, n = 5).

#### Matrigel plug angiogenesis assay

We used Matrigel plug angiogenesis assay (Corning, NY, USA) to evaluate the *in vivo* anti-angiogenic effect of TTAC-0001. It is based on the fact that VEGF secreted from Matrigel tumor cells exerts pro-angiogenic effects on the surrounding tissue, thus promoting neovascular development that is visualized in the Matrigel. If a drug inhibits the effect of VEGF by inhibiting either VEGF or VEGFR, then the decreased levels of neovascular development in the Matrigel can be evaluated.

Matrigel (0.5 mL) was premixed with 5 × 10^6^ MDA-MB-231 cells and then engrafted into the right mammary fat pad of Balb/c nude mice (n = 5). A single 2 or 10 mg/kg treatment of TTAC-0001 or saline was administered to the respective groups by intravenous injection after implantation. After 10 days, Matrigel plugs were removed and frozen for immunofluorescence analysis. To measure the hemoglobin (Hb) content, excised plugs (n = 5 plugs/group) were cut into small pieces and placed in 500 uL of cold, distilled water at 4°C overnight to liquefy the Matrigel. Specimens were centrifuged at 1500 rpm for 20 mins, and the supernatant was collected. Hb content was quantified using a Hb assay kit (Sigma-Aldrich) and spectrophotometry.

#### Immunofluoroscence staining

Cryosections (30 um) of the removed plugs were obtained. Plugs were then immunostained with an anti-mouse CD31 monoclonal antibody (1:300, Abcam, Cambridge, MA, UK) and a secondary goat anti-rabbit Alexa 594 antibody (1:5000, Life Technologies, Carlsbad, CA, USA), according to the manufacturer’s protocol.

The immunofluorescence images were analyzed using NIH-Image J software (National Institutes of Health, Bethesda, Maryland, USA). A free-hand ROI was drawn around the tumor, and CD31-positive cells were selected using the global histogram-derived thresholding method [19]. The percentage area of CD31-positive cells was calculated by dividing the area of the CD31-positive cells by the area of the tumor ROI.

### Anti-tumor efficacy

To evaluate the anti-tumor efficacy of the drugs, we used two types of cells and imaging methods as follows: (1) MDA-MB-231 cells for ultrasonography (US) evaluation and (2) MDA-MB-231+luc cells for bioluminescence imaging (BLI). The reason why we used imaging for tumor monitoring is that the orthotopic tumors in the mammary fat pad may be barely visible, thus making it very difficult to measure tumor size if the tumor decreases during treatment. In addition, utilization of both US and BLI modalities allows a more accurate evaluation of the tumor volume and viability, respectively.

#### Experimental groups

To evaluate the anti-tumor efficacy of the drugs, tumors were engrafted into 80 mice. Of these, 56 mice (70%) that met the tumor volume criteria (70–130 mm^3^) were selected and randomly assigned to four groups, which were treated with saline (control group), bevacizumab 10 mg/kg (BVZ-10 group), low-dose (2 mg/kg) TTAC-0001 (TTAC-2 group), and high-dose (10 mg/kg) TTAC-0001 (TTAC-10 group).

Mice were monitored daily for up to 30 days after treatment for tumor volume, body weight, and general body condition, such as appearance, food/water intake, respiration, and ambulation. Animals were euthanized when they showed signs of distress, when the tumors were >2 cm in diameter, when the weight loss was >15% of body weight, or when the tumor interfered with the ability to eat or drink.

#### Ultrasonography

All US examinations were performed by a board-certified radiologist (K.W.K) using an iU22 unit (Philips Healthcare, Bothell, WA, USA) with a 20 MHz linear transducer. To assess tumor volume, we measured the longest diameter of the tumor on the axial and coronal axes and the height of the tumor every 3 days during the 1 month follow-up. We calculated the tumor volume according to the following formula: 4.19 × axial longest diameter × coronal longest diameter × height.

#### Bioluminescence imaging

BLI is an established in vivo imaging techniques assessing assessing angiogenesis and evaluating the efficacy of angiogenesis-directed therapies [20, 21]. BLI was performed using the IVIS Lumina II machine (PerkinElmer, Waltham, MA, USA). Mice were anesthetized with 1% isoflurane (Forane®, Choongwae, Korea), then D-Luciferin (Caliper Life Sciences, Hopkinton, MA, USA) dissolved in phosphate-buffered saline (PBS; 1.5 mg luciferin/100 μL PBS) was injected intraperitoneally at a dose of 150 mg luciferin/kg, and serial images were acquired with an exposure time of 10 s, an f/stop of 1, and pixel binning of 8, over 20 min. The BLI was performed at baseline (i.e., 2 h before injecting therapeutic agents) and after treatment (every 5 days during the 1 month follow-up). Using Living Image® 4.2 software (Caliper Life Sciences, Hopkinton, MA, USA), we measured the total flux (photons/s) of the tumor bioluminescence signal by placing an ROI at the tumor.

#### Histopathologic study

All animals were euthanized at day 30 after treatment. The extracted tumors were perfused with PBS and fixed with 4% paraformaldehyde in PBS. Tumors were then embedded in paraffin and sectioned at a thickness of 5 μm at the largest tumor area.

To evaluate the tumor morphology and extent of viable tumor, hematoxylin and eosin (H&E) staining was performed. In representative sections of the tumor, H&E images were analyzed using NIH-Image J software. The viable tumor cells were regarded as cells stained with H&E in both the nucleus and cytoplasm, whereas necrotic/apoptotic areas were regarded as cells stained with eosin only or no stain. After drawing ROIs around the whole tumor, viable cells were selected based on the hematoxylin staining of the nuclei using the global histogram-derived thresholding method [19]. The percentage of viable tumor area was calculated by dividing the area of viable cells by the whole tumor area.

To evaluate the microvessel areas, immunohistochemical staining for a vascular endothelial antigen, CD31, was performed using a primary rabbit anti-mouse CD31 antibody (BD Pharmingen, San Diego, CA, USA) and a secondary goat anti-rabbit antibody (1:1000, Molecular Probes, Eugene, OR, USA). The images of immunohistochemical stains were analyzed using NIH-Image J software to calculate microvessel area. In the tumor ROI, microvessels were selected based on areas of CD31-positive cells [19]. The percentage microvessel area was calculated by dividing the area of CD31-positive cells by the area of the tumor ROI.

To evaluate tumor cell proliferation (i.e., the Ki-67 index), immunohistochemical stating for Ki-67 was performed using an anti-mouse Ki-67 antibody (1:100, Novus Biologicals, CO, USA). Detection was performed by incubating with the Dako EnVision + System HRP-labeled polyclonal anti-rabbit antibody (Agilent Inc., Palo Alto, CA, USA) for 30 min, followed by DAB chromogen (Agilent Inc.). In the tumor ROI, the Ki-67-positive cells were selected based on brownish nuclear staining using the global histogram-derived thresholding method [19]. We set the threshold to select the hematoxylin-stained nuclei and count the number of whole tumor cells. Then we reset the color threshold to select the brownish nuclei and count the number of Ki-67-stained tumor cells. The Ki-67 index was calculated by dividing the number of Ki-67-positive cells by the number of whole tumor cells.

### Statistical analyses

For the Matrigel plug angiogenesis assay, Hb content and percentage area of CD31-positive cells in the four different treatment groups were compared using one-way ANOVA with a post-hoc t-test with least significant difference significance (LSD).

For US and BLI analyses, the tumor volume on US and signal intensity on BLI over time (every 3 days on USI and every 5 days on BLI) using a repeated measures analysis of variance and post-hoc comparison tests with LSD were used. In addition, we compared the mean values of the tumor volume on US and signal intensity on BLI at 30 days in the four different treatment groups using one-way ANOVA with a post-hoc t-test with LSD.

For histopathologic analysis, we compared the percentage viable tumor area, percentage microvessel area, and Ki-67 index at 30 days in the four different treatment groups using one-way ANOVA with a post-hoc t-test with LSD.

Statistical analysis was performed using a computer software package (SPSS, version 21.0; SPSS; Chicago, IL, USA).

## Results

### Biodistribution for tumor targeting

**Fig 2:**
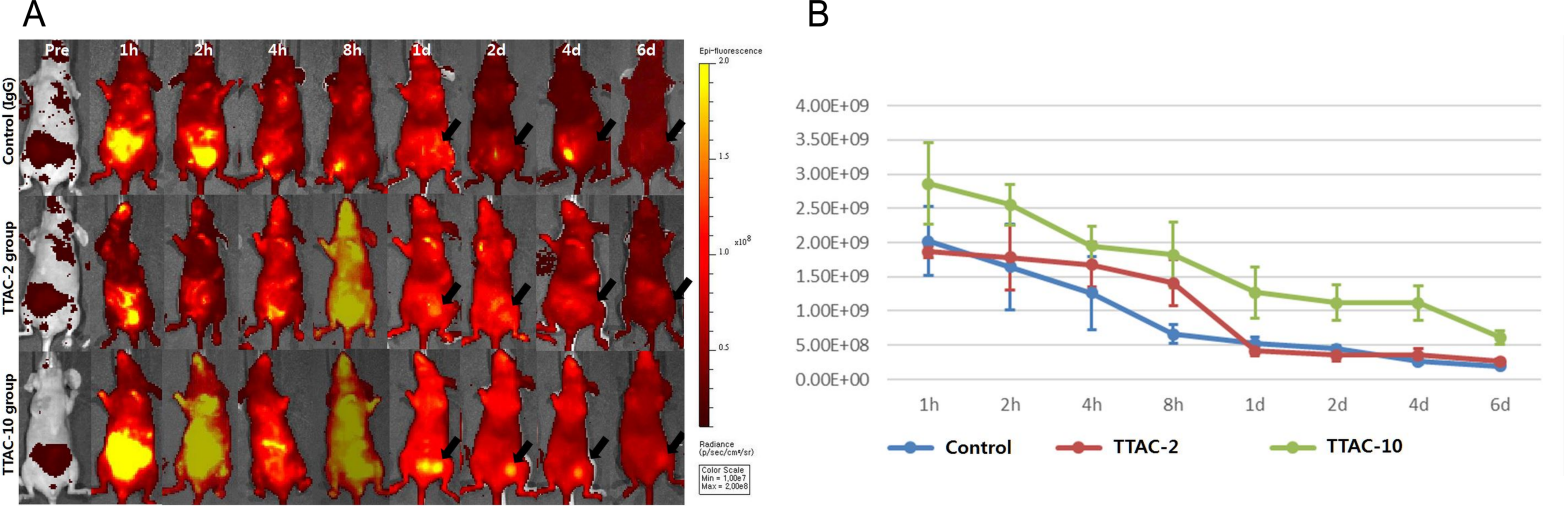
Biodistribution of TTAC-0001 using Alexa Fluor® 647 reactive dye labeling. (A) Serial fluorescence images were acquired at baseline and at 1, 2, 4, and 8 h and 1, 2, 4, and 6 days after drug injection. (B) Plots of fluorescence accumulation at the tumor implantation site for each group show the serial changes over time. TTAC-2, TTAC-0001 2 mg/kg; TTAC-10, TTAC-0001 10 mg/kg.

After injecting Alexa Fluor® 647-labeled TTAC-0001, a focal signal appeared in the right flank area after 24 h and was detected in both TTAC-2 and TTAC-10 groups, but not in the control group. These results are suggestive of tumor targeting of TTAC-0001 to the right mammary fat pad of the mouse. The TTAC-10 group demonstrated higher fluorescence accumulation at the tumor implantation site and a longer fluorescence accumulation of up to 6 days than TTAC-2 group (Fig 2), indicating a higher and longer level of tumor targeting in the high-dose TTAC-10 group.

### Mode of action of the anti-angiogenic effect

**Fig 3:**
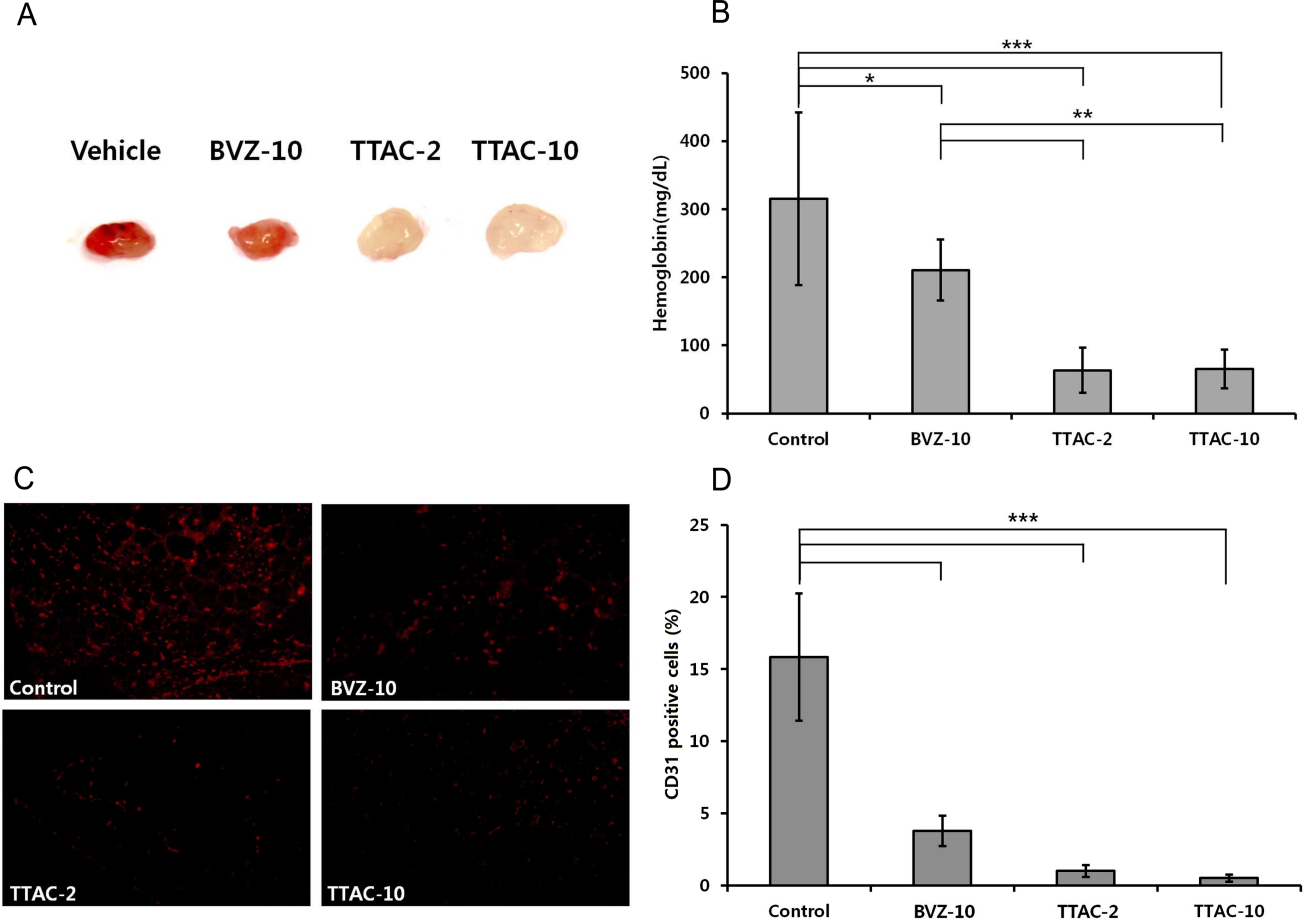
Anti-angiogenic activity of TTAC-0001 in the Matrigel plug assay. (A) Gross overview of Matrigel plugs and (B) hemoglobin content (mean ± sd, n = 5). (C) Immunohistochemical images showing CD31-positive blood vessels (red) in the Matrigel plug. Scale bars; 200 um. (D) Density of CD31-positve blood vessels (mean ± sd) in the Matrigel plugs. ***, *P* < 0.001; **, *P* < 0.01; *, *P* < 0.05. BVZ-10, bevacizumab 10 mg/kg; TTAC-2, TTAC-0001 2 mg/kg; TTAC-10, TTAC-0001 10 mg/kg.

Matrigel plugs from TTAC-0001-treated groups were pale white in appearance, but those from the control group were bright red (Fig 3), indicating a reduction in new blood vessel formation in the TTAC-0001-treated groups. The Hb content in the Matrigel plugs was the highest in the control group, followed by BVZ-10, TTAC-2, and TTAC-10 groups. A significant difference was noted between both TTAC-0001-treated groups (TTAC-2 and TTAC-10 groups) and the BVZ-10 group (*P* = 0.004 and *P* = 0.004, Fig 3). However, there was no significant difference between the TTAC-2 and TTAC-10 Matrigel plugs (*P* = 0.971, Fig 3).

The percentage area of CD31-positive cells showed a similar tendency in that the TTAC-2 and TTAC-10 groups had a significantly reduced area of CD31-positive cells compared with the control group and the BVZ-10 group (*P* < 0.05, Fig 3). These results consistently indicated that the mode of action of TTAC-0001 was an anti-angiogenetic effect, which was the strongest in the TTAC-10 group, followed by the TTAC-2 and BVZ-10 groups.

### Anti-tumor efficacy

Of the 56 mice involved in the anti-tumor efficacy evaluation, none were found dead during the 30-day treatment period. No significant differences in weights were seen between the four groups in the US and BLI experiments (S1 Fig and S2 Fig). Signs of drug toxicity, such as ruffled fur, anorexia, cachexia, skin tenting, skin ulcerations, or toxic death [22], were not seen in any of the mice.

#### Serial US monitoring

**Fig 4:**
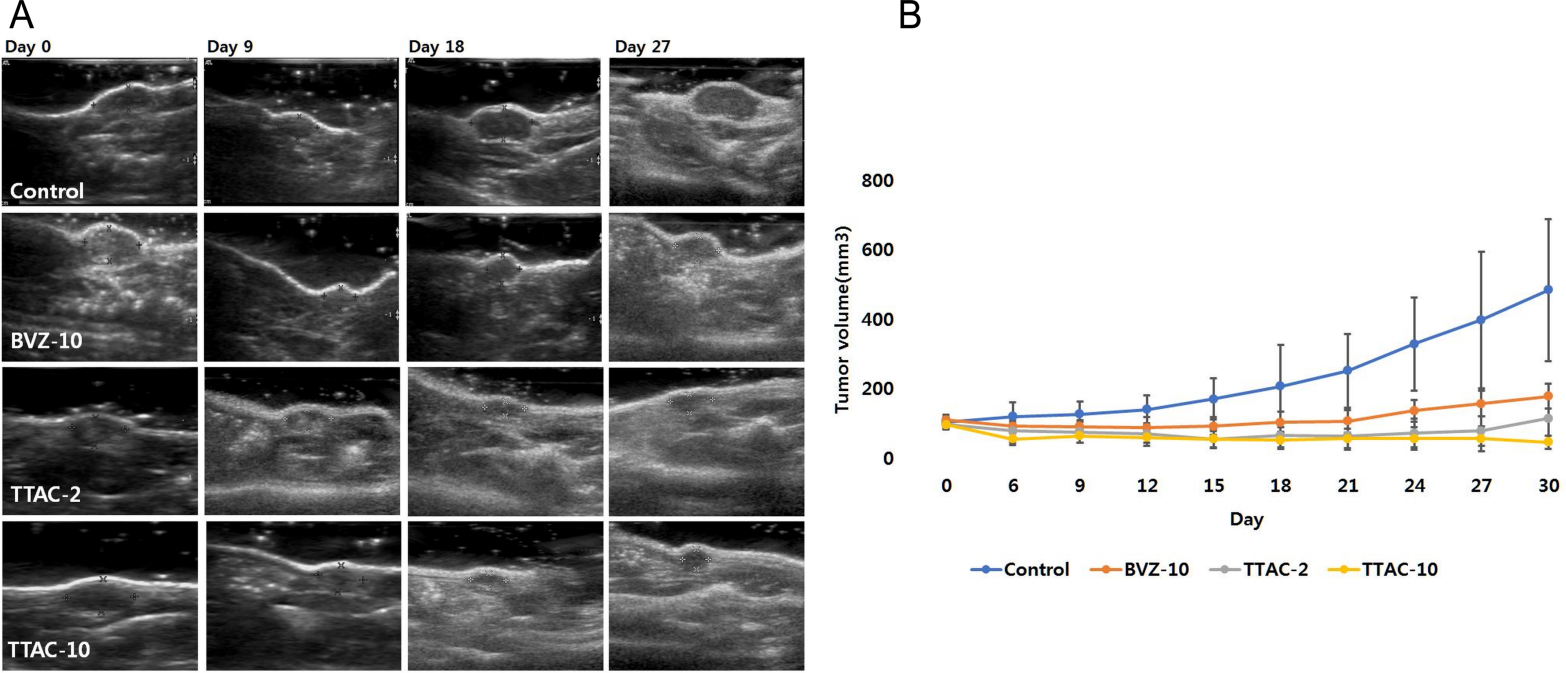
Serial ultrasonography images of mouse breast cancer before and after anti-angiogenic treatment. (A) The longest diameter of the tumor on the axial and coronal axes and the height of the tumor were measured using ultrasonography. (B) Plots of tumor volumes for each group show the serial changes over the treatment period. Data are presented as mean ± sd in the graphs. BVZ-10, bevacizumab 10 mg/kg; TTAC-2, TTAC-0001 2 mg/kg; TTAC-10, TTAC-0001 10 mg/kg.

Serial changes in the mean tumor volume in mice transplanted with MDA-MB-231 cells were measured every 3 days and are summarized in Table 2. The control group showed a continuous increase in tumor volume over time. In contrast, all treated groups (BVZ-10, TTAC-2, and TTAC-10) showed significantly smaller mean tumor volumes (*P* < 0.001, Fig 4). The efficacy of tumor growth inhibition was the strongest in the TTAC-10 group, followed by the TTAC-2 and BVZ-10 groups (*P* = 0.018), but the TTAC-10 group did not differ significantly from the TTAC-2 group (*P* = 0.182).

**Table 2:** Serial changes in the mean tumor volume from ultrasonography imaging in mice treated with bevacizumab or TTAC-0001.

	*Tumor volume (mm*^*3*^*)*			
*Time point*	Control group*	*BVZ-10*	*TTAC-2*	*TTAC-10*
0 days	102.5 ± 15.5	108.8 ± 14.1	95.2 ± 12.9	95.9 ± 11.1
6 days	118.8 ± 40.2	90.8 ± 18.1	78.0 ± 33.7	53.7 ± 17.3
9 days	125.6 ± 35.4	90.2 ± 31.0	73.7 ± 29.9	63.3 ± 19.3
12 days	139.6 ± 40.0	86.9 ± 26.7	68.4 ± 25.3	59.0 ± 25.0
15 days	169.1 ± 59.3	91.6 ± 31.4	54.7 ± 27.5	54.4 ± 23.6
18 days	206.1 ± 118.8	101.7 ± 32.6	64.7 ± 33.6	50.6 ± 24.6
21 days	250.6 ± 106.6	105.3 ± 31.1	63.1 ± 40.6	55.4 ± 27.3
24 days	327.8 ± 133.5	135.9 ± 37.3	70.9 ± 41.6	55.4 ± 32.7
27 days	397.0 ± 196.6	155.9 ± 35.6	77.5 ± 42.9	54.9 ± 36.6
30 days	482.8 ± 204.4	177.0 ± 34.0	113.9 ± 64.9	44.9 ± 18.9

*Treated with saline.

BVZ-10, bevacizumab 10 mg/kg; TTAC-2, TTAC-0001 2 mg/kg; TTAC-10, TTAC-0001 10 mg/kg

#### Serial BLI monitoring

**Fig 5:**
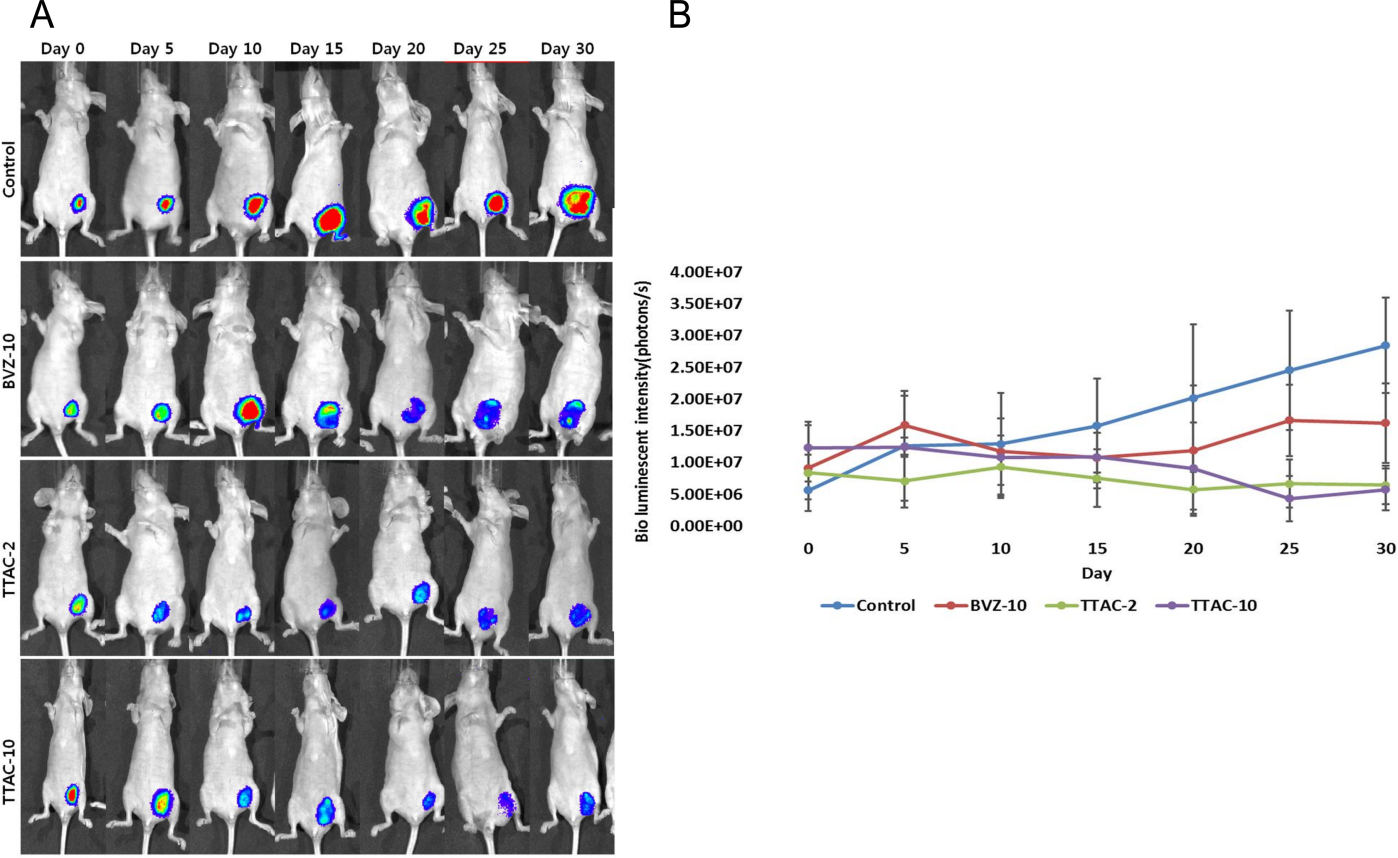
Serial bioluminescence imaging of mouse breast cancer before and after anti-angiogenic treatment. (A) The bioluminescence imaging signal was measured at baseline and every 5 days after the administration of anti-angiogenic agents. (B) Plots of tumor volumes for each group show the serial changes over the treatment period. Data are presented as mean ± sd in the graphs. BVZ-10, bevacizumab 10 mg/kg; TTAC-2, TTAC-0001 2 mg/kg; TTAC-10, TTAC-0001 10 mg/kg.

The BLI signal intensities of MDA-MB-231+luc cell tumors showed different characteristics between the groups, as summarized in Table 3. In the control group, the signal intensity of tumors continuously increased until 30 days. In the BVZ-10 group, the signal intensity of the tumors fluctuated during the treatment period (Fig 5). In both TTAC-2 and TTAC-10 groups, the signal intensity of tumors showed a tendency to decrease. Notably, the TTAC-10 group showed a rapid drop in the signal intensity of tumors after 20 days (Fig 5).

**Table 3:** Serial changes in the mean signal intensity from bioluminescence imaging of mice treated with bevacizumab or TTAC-0001.

	*Signal intensity of tumor*			
*Time point*	Control group*	*BVZ-10*	*TTAC-2*	*TTAC-10*
0 days	0.56 × 10^7^ ± 0.14 × 10^7^	0.99 × 10^7^ ± 0.67 × 10^7^	0.84 × 10^7^ ± 0.29 × 10^7^	1.24 × 10^7^ ± 0.41 × 10^7^
5 days	1.27 × 10^7^ ± 0.87 × 10^7^	1.36 × 10^7^ ± 0.46 × 10^7^	0.71 × 10^7^ ± 0.42 × 10^7^	1.24 × 10^7^ ± 0.15 × 10^7^
10 days	1.29 × 10^7^ ± 0.80 × 10^7^	1.12 × 10^7^ ± 0.52 × 10^7^	0.94 × 10^7^ ± 0.49 × 10^7^	1.08 × 10^7^ ± 0.61 × 10^7^
15 days	1.58 × 10^7^ ± 0.74 × 10^7^	1.00 × 10^7^ ± 0.39 × 10^7^	0.75 × 10^7^ ± 0.45 × 10^7^	1.09 × 10^7^ ± 0.49 × 10^7^
20 days	2.02 × 10^7^ ± 1.17 × 10^7^	1.13 × 10^7^ ± 1.03 × 10^7^	0.58 × 10^7^ ± 0.32 × 10^7^	0.91 × 10^7^ ± 0.72 × 10^7^
25 days	2.46 × 10^7^ ± 0.94 × 10^7^	1.20 × 10^7^ ± 0.56 × 10^7^	0.67 × 10^7^ ± 0.38 × 10^7^	0.44 × 10^7^ ± 0.36 × 10^7^
30 days	2.85 × 10^7^ ± 0.75 × 10^7^	1.27 × 10^7^ ± 0.62 × 10^7^	0.65 × 10^7^ ± 0.30 × 10^7^	0.58 × 10^7^ ± 0.33 × 10^7^

*Treated with saline.

BVZ-10, bevacizumab 10 mg/kg; TTAC-2, TTAC-0001 2 mg/kg; TTAC-10, TTAC-0001 10 mg/kg

At the end of the treatment period (30^th^ day), the efficacy of tumor inhibition was the strongest in the TTAC-10 group, followed by the TTAC-2 and BVZ-10 groups (*P* = 0.040), but the TTAC-10 group did not differ from TTAC-2 group (*P* = 0.831).

#### Histopathologic findings

**Fig 6:**
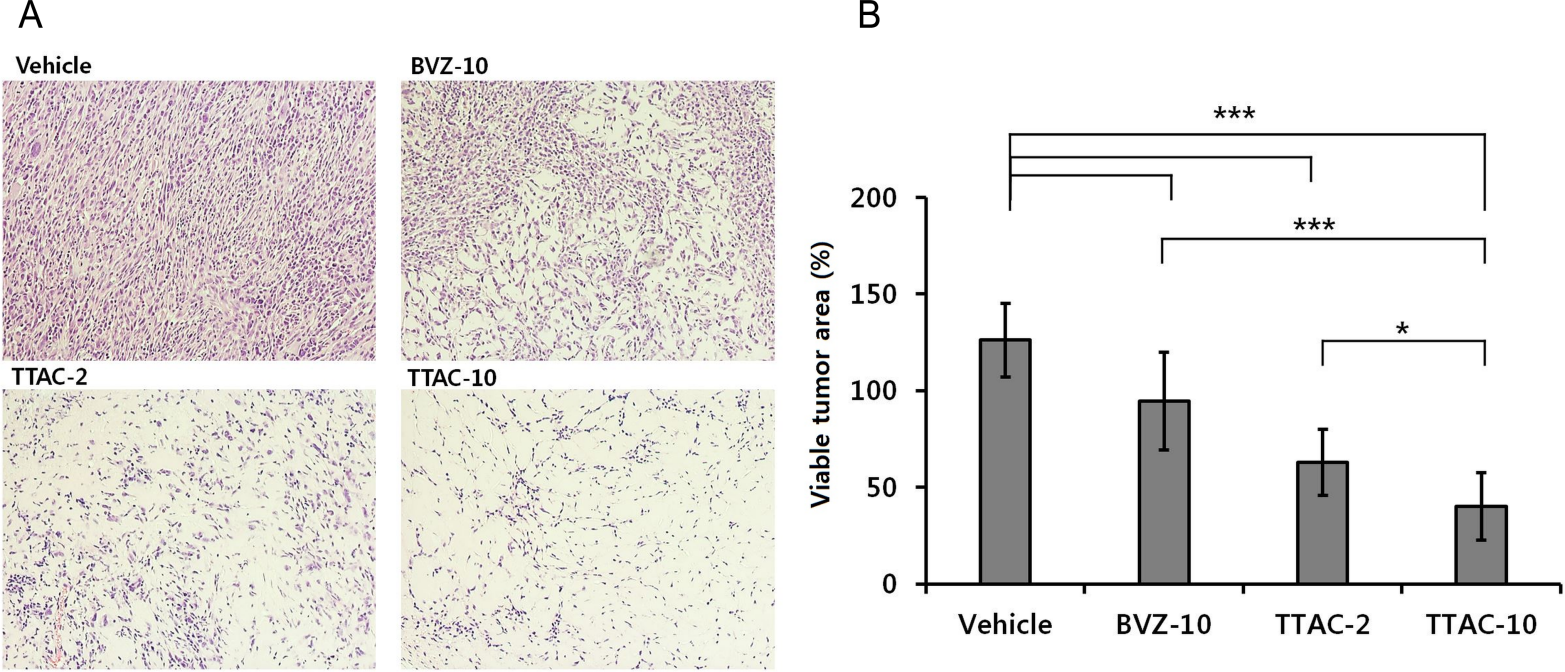
Histopathologic specimens with hematoxylin and eosin staining. (A) Microscope images (magnification × 200) of the four groups (control, BVZ-10, TTAC-2, TTAC-10). (B) The viable areas in tumors are shown for each group. Data are presented as mean ± sd in the graphs. ***, *P* < 0.001; **, *P* < 0.01; *, *P* < 0.05. BVZ-10, bevacizumab 10 mg/kg; TTAC-2, TTAC-0001 2 mg/kg; TTAC-10, TTAC-0001 10 mg/kg.

H&E staining demonstrated different characteristics of tumor apoptosis/necrosis between the four groups (Fig 6). Tumor cells with active mitosis were abundant in the control group, whereas they were sparse in the treatment groups. The percentage of viable tumor areas significantly differed between groups (*P* < 0.001, one-way ANOVA) (Fig 6). The post-hoc test showed that the percentage of viable tumor area was the lowest in the TTAC-10 group, followed by the TTAC-2 and BVZ-10 groups, with a significant difference in each pair (*P* ≤ 0.016).

**Fig 7:**
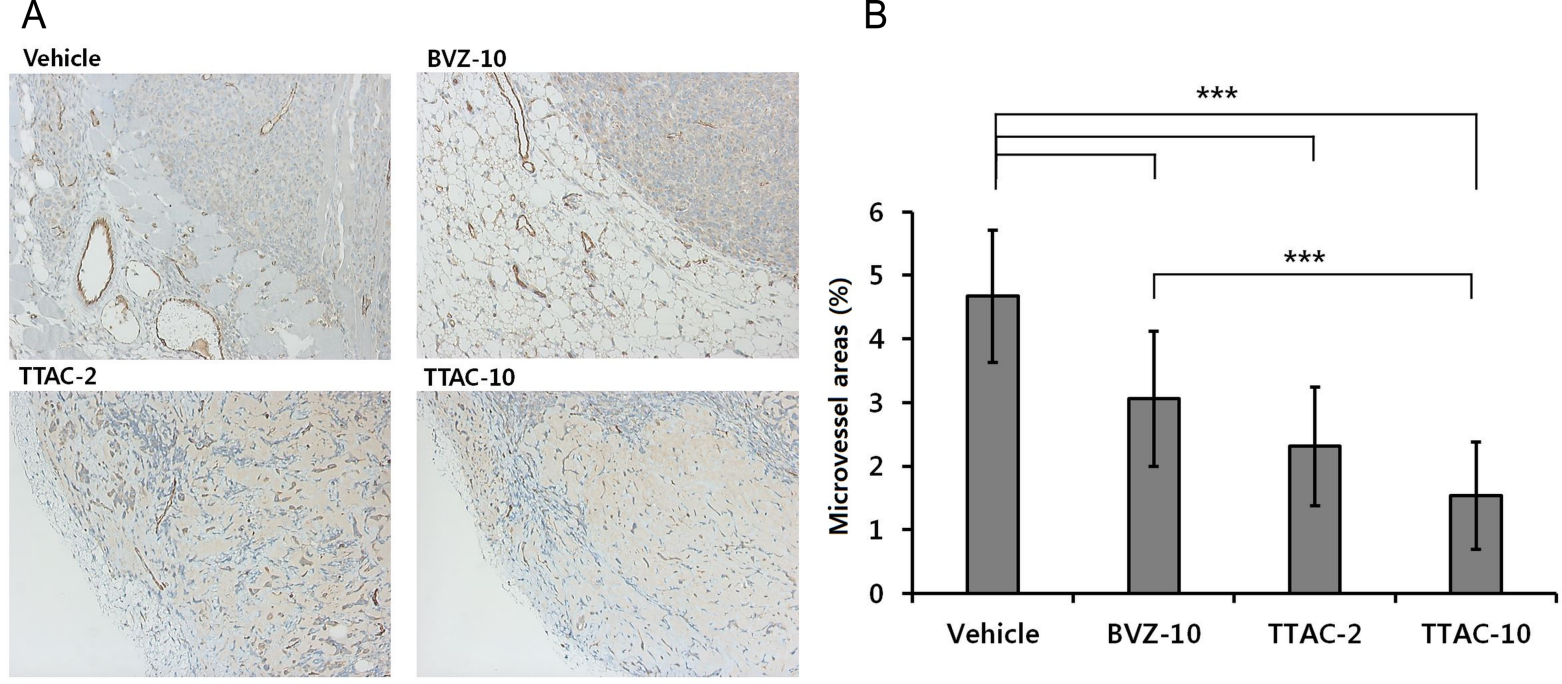
Immunohistochemistry with the anti-CD31 antibody for blood vessel detection. (A) Microscope images (magnification × 200) of the four groups (control, BVZ-10, TTAC-2, TTAC-10). (B) The vessel density in the tumors is shown for each group. Data are presented as mean ± sd in the graphs. ***, *P* < 0.001; **, *P* < 0.01; *, *P* < 0.05. BVZ-10, bevacizumab 10 mg/kg; TTAC-2, TTAC-0001 2 mg/kg; TTAC-10, TTAC-0001 10 mg/kg.

Immunohistochemical staining for CD31 showed that tumor vessels were abundant in the control group, but sparse in treated groups, with smaller microvessel areas (Fig 7). One-way ANOVA revealed that the percentage of microvessel area significantly differed between the four groups (*P* < 0.001). The post-hoc test revealed that the TTAC-10 group showed the lowest percentage of microvessel area, followed by the TTAC-2 (*P* = 0.001) and BVZ-10 groups. However, there was no significant difference between the TTAC-2 and TTAC-10 groups (*P =* 0.084).

**Fig 8:**
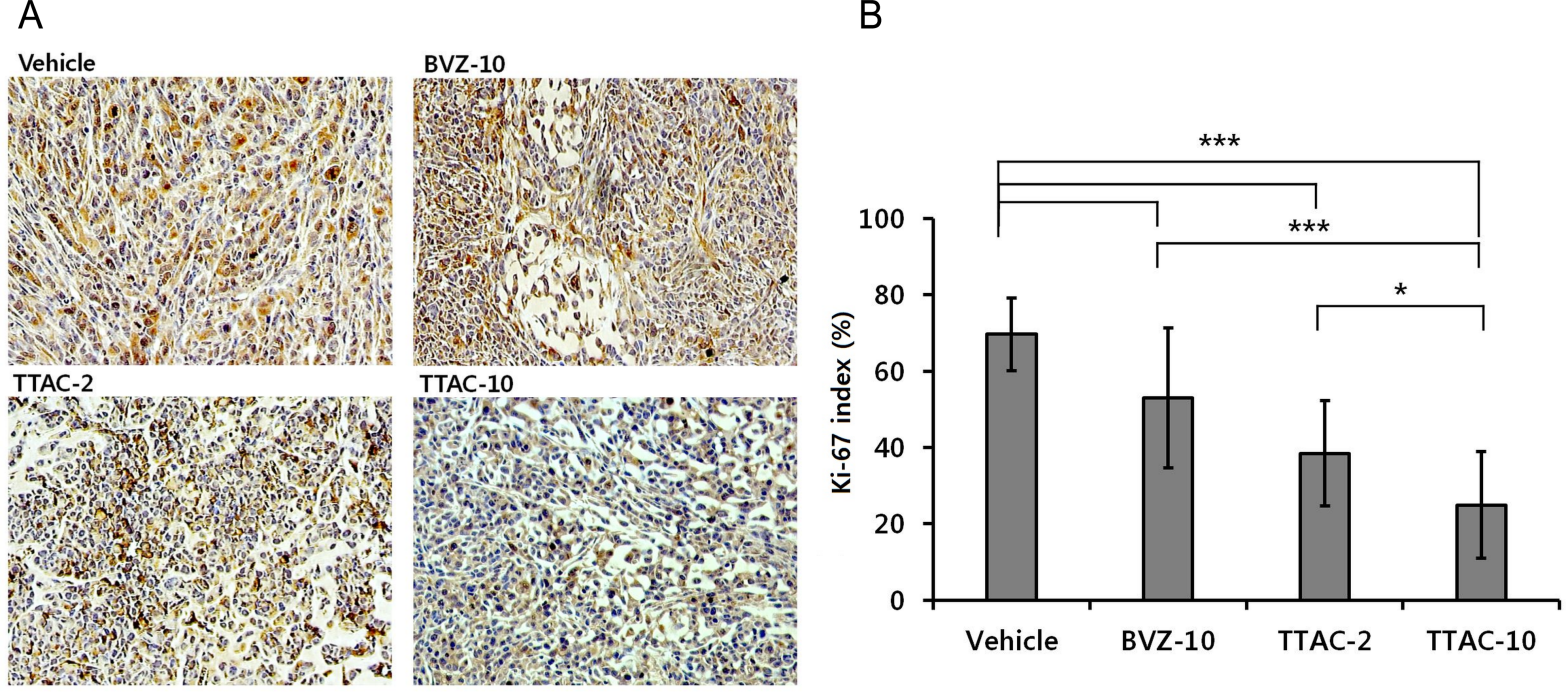
Immunohistochemistry with the anti-Ki-67 antibody to detect proliferation. (A) Microscope images (magnification × 400) of the four groups (control, BVZ-10, TTAC-2, TTAC-10). (B) The Ki-67-positive cells in tumors are shown for each group. Data are presented as mean ± sd in the graphs. ***, *P* < 0.001; **, *P* < 0.01; *, *P* < 0.05. BVZ-10, bevacizumab 10 mg/kg; TTAC-2, TTAC-0001 2 mg/kg; TTAC-10, TTAC-0001 10 mg/kg.

Immunohistochemical staining with Ki-67 demonstrated that cell proliferation markedly decreased in both TTAC-2 and TTAC-10 groups (Fig 8). One-way ANOVA revealed that the Ki-67 indexes significantly differed between the four groups (*P* < 0.001). The post-hoc test revealed that the TTAC-10 group showed the lowest Ki-67 index, followed by TTAC-2 and BVZ-10 groups, with a significant difference between each pair (*P* ≤ 0.041).

## Discussion

In our study, TTAC-0001, a novel fully human anti-VEGFR-2/KDR monoclonal antibody that blocks VEGF/VEGFR-2 signaling, showed selective tumor targeting that lasted at least 6 days, an anti-angiogenic effect to inhibit neovascularization, and anti-tumor efficacy. Among the three treatment groups, TTAC-10 showed the highest level of tumor targeting, anti-angiogenic effects, and anti-tumor efficacy, compared with the TTAC-2 and the BVZ-10 groups. The anti-tumor efficacy for inhibition of tumor growth was similar between the TTAC-2 and BVZ-10 groups. In histopathologic examination, the TTAC-10 group showed the lowest viable tumor area, microvessel area, and cellular proliferation among the treatment groups. In addition, no significant toxicity or death was noted for all treated mice. Consistent with a previous report [8], our results indicate the validity of TTAC-0001 10 mg/kg as a potent anti-angiogenic agent.

Targeting VEGFR-2/KDR may be more effective than targeting the VEGF ligand itself. Considering the fact that other VEGF-like heparin-binding growth factors can prevent specific inhibition of the anti-VEGF antibody in hypoxic tumor microenvironments, targeting VEGFR-2/KDR is more effective for anti-angiogenesis [8, 23, 24]. VEGFR-2/KDR is more easily targetable than VEGF because VEGFR-2/KDR is highly expressed on the surface of the activated endothelium in tumor tissues, in contrast to VEGF, which is mainly located in the interstitial space between cells. Therefore, the VEGFR-2 antibody can more effectively inhibit neo-angiogenesis [23, 24]. Moreover, different acting mechanisms of anti-VEGFR-2 antibody which inhibits the binding of VEGFR2 and all of VEGF-A, VEGF-C, and VEGF-D compared to VEGF antagonist, which only inhibits VEGF-A activity may contribute the increased anti-angiogenic efficacy of TTAC-0001 [25]. These findings support our results showing that TTAC-0001 10 mg/kg has a more potent anti-angiogenic effect and anti-tumor efficacy than bevacizumab 10 mg/kg.

Preclinical results do not guarantee the success of clinical trials. However, the more information obtained in the preclinical drug development stage, the greater the probability of success in clinical trials [26]. As the researchers recognized the values of information from the imaging experiments, utilization of various imaging modalities has become increased. In our study, we showed the anti-tumor efficacy by using an orthotopic tumor model and US/BLI imaging methods, through which we expect to increase the translatability of our results to clinical trials [27]. Moreover, validation of targeting capacity of TTAC-0001 confirmed by the biodistribution exams can be used as a reference for predicting the mode of action and therapeutic efficacy of TTAC-0001 in humans as the TTAC-0001 has cross-reactivity between human and murine species [7]. It is expected that TTAC-0001 can binds to the N-terminus of these domains in the extracellular region of either human or mice KDR [8]. Although bevacizumab has no cross-reactivity between human and murine species, previous studies demonstrated anti-angiogenic effect in human cancer cell line xenografts [28, 29].

Although the anti-tumor activity of TTAC-0001 has been shown in colorectal, non-small-cell lung cancer [8–10], our study is the first study to demonstrate anti-tumor activity of TTAC-0001 in triple-negative breast cancer. In contrast to previous studies, as we used orthotopic xenograft tumor model, the assessment of anti-tumor activity is more reliable than other tumor models, as the orthotopic xenograft models have similar tumor microenvironment as the original tumor [30, 31]. In addition, we first showed the drug mechanism and the anti-tumor efficacy of the TTAC-0001 using bioimaging methods.

In patients with triple-negative breast cancer, anti-angiogenic agents, such as bevacizumab and ramucirumab, have not been successful [32]. In particular, bevacizumab, the first approved anti-angiogenic agent to bind all isoforms of VEGF-A, was approved for metastatic, HER2-negative breast cancer in 2008, based on the E2100 trial in which the progression-free survival was significantly improved [13, 33]. However, subsequent trials did not show that improvement of progression-free survival leads to an overall survival gain in patients with metastatic breast cancer [34, 35]. Although data from basic and translational science and the clinical success in improving progression-free survival may imply the effectiveness of anti-angiogenic therapy for inhibition of tumor growth, anti-angiogenic agents might be effective only in a proportion of breast cancer patients as the breast cancer is regarded as a heterogenous group of tumors [36]. The next step may include the development of a more potent anti-angiogenic agent covering more targets, and the selective use of anti-angiogenic agents in a selected population of breast cancer patients [11, 32, 36].

## Conclusions

In summary, TTAC-0001, a novel fully human monoclonal antibody against VEGFR-2/KDR, was shown to have anti-angiogenic effects and good anti-tumor efficacy for inhibition of tumor growth and neovascularization in a mouse orthotopic triple-negative breast cancer model. These preclinical results may assist translation into clinical investigation of TTAC-0001 for breast cancer patients.

## Supporting information

S1 Fig Monitoring of mice body weight during the 30-day treatment period in the ultrasonography experiment. Body weight of the four groups (control, bevacizumab 10 mg/kg; TTAC-0001 2 mg/kg; TTAC-0001 10 mg/kg) was monitored prior to any treatment (0 day) and then every 3 days.
(TIF)

S2 Fig Monitoring of mice body weight during the 30-day treatment period in the bioluminescence imaging experiment. Body weight of the four groups (control, bevacizumab 10 mg/kg; TTAC-0001 2 mg/kg; TTAC-0001 10 mg/kg) was monitored prior to any treatment (0 day) and then every 5 days.
(TIF)

## References

[1] Weidner N. Tumour vascularity and proliferation: clear evidence of a close relationship. J Pathol. 1999;189(3):297–9. Epub 1999/11/05. doi: 10.1002/(SICI)1096-9896(199911)189:3<297::AID-PATH434>3.0.CO;2-O . 10547589 10.1002/(SICI)1096-9896(199911)189:3<297::AID-PATH434>3.0.CO;2-O

[2] Fontanella C, Ongaro E, Bolzonello S, Guardascione M, Fasola G, Aprile G. Clinical advances in the development of novel VEGFR2 inhibitors. Annals of translational medicine. 2014;2(12):123 doi: 10.3978/j.issn.2305-5839.2014.08.14 ; PubMed Central PMCID: PMC4260048. 25568876 10.3978/j.issn.2305-5839.2014.08.14 PMC4260048

[3] Potente M, Gerhardt H, Carmeliet P. Basic and therapeutic aspects of angiogenesis. Cell. 2011;146(6):873–87. Epub 2011/09/20. doi: 10.1016/j.cell.2011.08.039 . 21925313 10.1016/j.cell.2011.08.039

[4] Hanahan D, Weinberg RA. Hallmarks of cancer: the next generation. Cell. 2011;144(5):646–74. Epub 2011/03/08. doi: 10.1016/j.cell.2011.02.013 . 21376230 10.1016/j.cell.2011.02.013

[5] Zhu Z, Bohlen P, Witte L. Clinical development of angiogenesis inhibitors to vascular endothelial growth factor and its receptors as cancer therapeutics. Curr Cancer Drug Targets. 2002;2(2):135–56. Epub 2002/08/22. . 12188915 10.2174/1568009023333881

[6] Lee SH. Tanibirumab (TTAC-0001): a fully human monoclonal antibody targets vascular endothelial growth factor receptor 2 (VEGFR-2). Archives of pharmacal research. 2011;34(8):1223–6. doi: 10.1007/s12272-011-0821-9 . 21910042 10.1007/s12272-011-0821-9

[7] Lee WS, Pyun BJ, Kim SW, Shim SR, Nam JR, Yoo JY, et al TTAC-0001, a human monoclonal antibody targeting VEGFR-2/KDR, blocks tumor angiogenesis. MAbs. 2015;7(5):957–68. Epub 2015/05/06. doi: 10.1080/19420862.2015.1045168 ; PubMed Central PMCID: PMCPMC4622656. 25942475 10.1080/19420862.2015.1045168 PMC4622656

[8] Kim DG, Jin Y, Jin J, Yang H, Joo KM, Lee WS, et al Anticancer activity of TTAC-0001, a fully human anti-vascular endothelial growth factor receptor 2 (VEGFR-2/KDR) monoclonal antibody, is associated with inhibition of tumor angiogenesis. MAbs. 2015;7(6):1195–204. Epub 2015/09/02. doi: 10.1080/19420862.2015.1086854 ; PubMed Central PMCID: PMCPMC4966428. 26325365 10.1080/19420862.2015.1086854 PMC4966428

[9] Lee SJ, Lee SY, Lee WS, Yoo JS, Sun JM, Lee J, et al Phase I trial and pharmacokinetic study of tanibirumab, a fully human monoclonal antibody to vascular endothelial growth factor receptor 2, in patients with refractory solid tumors. Invest New Drugs. 2017 Epub 2017/04/10. doi: 10.1007/s10637-017-0463-y . 28391576 10.1007/s10637-017-0463-y PMC5694508

[10] Tampellini M, Sonetto C, Scagliotti GV. Novel anti-angiogenic therapeutic strategies in colorectal cancer. Expert Opin Investig Drugs. 2016;25(5):507–20. Epub 2016/03/05. doi: 10.1517/13543784.2016.1161754 . 26938715 10.1517/13543784.2016.1161754

[11] Mancini P, Angeloni A, Risi E, Orsi E, Mezi S. Standard of care and promising new agents for triple negative metastatic breast cancer. Cancers. 2014;6(4):2187–223. doi: 10.3390/cancers6042187 ; PubMed Central PMCID: PMC4276962. 25347122 10.3390/cancers6042187 PMC4276962

[12] Friedman HS, Prados MD, Wen PY, Mikkelsen T, Schiff D, Abrey LE, et al Bevacizumab alone and in combination with irinotecan in recurrent glioblastoma. J Clin Oncol. 2009;27(28):4733–40. Epub 2009/09/02. doi: 10.1200/JCO.2008.19.8721 . 19720927 10.1200/JCO.2008.19.8721

[13] Miller K, Wang M, Gralow J, Dickler M, Cobleigh M, Perez EA, et al Paclitaxel plus bevacizumab versus paclitaxel alone for metastatic breast cancer. The New England journal of medicine. 2007;357(26):2666–76. doi: 10.1056/NEJMoa072113 . 18160686 10.1056/NEJMoa072113

[14] Willmann JK, van Bruggen N, Dinkelborg LM, Gambhir SS. Molecular imaging in drug development. Nature reviews Drug discovery. 2008;7(7):591–607. Epub 2008/07/02. doi: 10.1038/nrd2290 . 18591980 10.1038/nrd2290

[15] Weber WA, Czernin J, Phelps ME, Herschman HR. Technology Insight: novel imaging of molecular targets is an emerging area crucial to the development of targeted drugs. Nature clinical practice Oncology. 2008;5(1):44–54. Epub 2007/12/22. doi: 10.1038/ncponc0982 ; PubMed Central PMCID: PMCPMC2830564. 18097456 10.1038/ncponc0982 PMC2830564

[16] Ayers GD, McKinley ET, Zhao P, Fritz JM, Metry RE, Deal BC, et al Volume of preclinical xenograft tumors is more accurately assessed by ultrasound imaging than manual caliper measurements. Journal of ultrasound in medicine: official journal of the American Institute of Ultrasound in Medicine. 2010;29(6):891–901. Epub 2010/05/26. ; PubMed Central PMCID: PMCPMC2925269. 20498463 10.7863/jum.2010.29.6.891 PMC2925269

[17] Dufort S, Sancey L, Wenk C, Josserand V, Coll JL. Optical small animal imaging in the drug discovery process. Biochimica et biophysica acta. 2010;1798(12):2266–73. Epub 2010/03/30. doi: 10.1016/j.bbamem.2010.03.016 . 20346346 10.1016/j.bbamem.2010.03.016

[18] El-Deiry WS, Sigman CC, Kelloff GJ. Imaging and oncologic drug development. J Clin Oncol. 2006;24(20):3261–73. Epub 2006/07/11. doi: 10.1200/JCO.2006.06.5623 . 16829650 10.1200/JCO.2006.06.5623

[19] Schneider CA, Rasband WS, Eliceiri KW. NIH Image to ImageJ: 25 years of image analysis. Nature methods. 2012;9(7):671–5. Epub 2012/08/30. . 22930834 10.1038/nmeth.2089 PMC5554542

[20] Virostko J, Jansen ED. Validation of bioluminescent imaging techniques. Methods in molecular biology (Clifton, NJ). 2009;574:15–23. Epub 2009/08/18. doi: 10.1007/978-1-60327-321-3_2 . 19685296 10.1007/978-1-60327-321-3_2

[21] Virostko J, Xie J, Hallahan DE, Arteaga CL, Gore JC, Manning HC. A molecular imaging paradigm to rapidly profile response to angiogenesis-directed therapy in small animals. Molecular imaging and biology: MIB: the official publication of the Academy of Molecular Imaging. 2009;11(3):204–12. Epub 2009/01/09. doi: 10.1007/s11307-008-0193-9 ; PubMed Central PMCID: PMCPmc2677126. 19130143 10.1007/s11307-008-0193-9 PMC2677126

[22] Baguley BC, Holdaway KM, Thomsen LL, Zhuang L, Zwi LJ. Inhibition of growth of colon 38 adenocarcinoma by vinblastine and colchicine: evidence for a vascular mechanism. Eur J Cancer. 1991;27(4):482–7. Epub 1991/01/01. . 1827725 10.1016/0277-5379(91)90391-p

[23] Brekken RA, Huang X, King SW, Thorpe PE. Vascular endothelial growth factor as a marker of tumor endothelium. Cancer research. 1998;58(9):1952–9. Epub 1998/05/15. . 9581838

[24] Zhu Z, Lu D, Kotanides H, Santiago A, Jimenez X, Simcox T, et al Inhibition of vascular endothelial growth factor induced mitogenesis of human endothelial cells by a chimeric anti-kinase insert domain-containing receptor antibody. Cancer Lett. 1999;136(2):203–13. Epub 1999/06/04. . 10355750 10.1016/s0304-3835(98)00324-3

[25] Witte L, Hicklin DJ, Zhu Z, Pytowski B, Kotanides H, Rockwell P, et al Monoclonal antibodies targeting the VEGF receptor-2 (Flk1/KDR) as an anti-angiogenic therapeutic strategy. Cancer metastasis reviews. 1998;17(2):155–61. Epub 1998/10/14. . 9770111 10.1023/a:1006094117427

[26] Kuhlmann J. Alternative strategies in drug development: clinical pharmacological aspects. International journal of clinical pharmacology and therapeutics. 1999;37(12):575–83. Epub 1999/12/22. . 10599949

[27] Rezaee R, Abdollahi M. The importance of translatability in drug discovery. Expert Opinion on Drug Discovery. 2017;12(3):237–9. doi: 10.1080/17460441.2017.1281245 28095719 10.1080/17460441.2017.1281245

[28] Michishita M, Ohtsuka A, Nakahira R, Tajima T, Nakagawa T, Sasaki N, et al Anti-tumor effect of bevacizumab on a xenograft model of feline mammary carcinoma. The Journal of veterinary medical science. 2016;78(4):685–9. Epub 2015/12/01. doi: 10.1292/jvms.15-0550 ; PubMed Central PMCID: PMCPmc4873862. 26616000 10.1292/jvms.15-0550 PMC4873862

[29] Liang WC, Wu X, Peale FV, Lee CV, Meng YG, Gutierrez J, et al Cross-species vascular endothelial growth factor (VEGF)-blocking antibodies completely inhibit the growth of human tumor xenografts and measure the contribution of stromal VEGF. The Journal of biological chemistry. 2006;281(2):951–61. Epub 2005/11/10. doi: 10.1074/jbc.M508199200 . 16278208 10.1074/jbc.M508199200

[30] Kocaturk B, Versteeg HH. Orthotopic injection of breast cancer cells into the mammary fat pad of mice to study tumor growth. Journal of visualized experiments: JoVE. 2015;(96). Epub 2015/03/06. doi: 10.3791/51967 ; PubMed Central PMCID: PMCPmc4354624. 25742185 10.3791/51967 PMC4354624

[31] Qiu W, Su GH. Development of orthotopic pancreatic tumor mouse models. Methods in molecular biology (Clifton, NJ). 2013;980:215–23. Epub 2013/01/30. doi: 10.1007/978-1-62703-287-2_11 ; PubMed Central PMCID: PMCPmc4049460. 23359156 10.1007/978-1-62703-287-2_11 PMC4049460

[32] Aalders KC, Tryfonidis K, Senkus E, Cardoso F. Anti-angiogenic treatment in breast cancer: Facts, successes, failures and future perspectives. Cancer treatment reviews. 2017;53:98–110. Epub 2017/01/15. doi: 10.1016/j.ctrv.2016.12.009 . 28088074 10.1016/j.ctrv.2016.12.009

[33] Miles DW, Chan A, Dirix LY, Cortes J, Pivot X, Tomczak P, et al Phase III study of bevacizumab plus docetaxel compared with placebo plus docetaxel for the first-line treatment of human epidermal growth factor receptor 2-negative metastatic breast cancer. J Clin Oncol. 2010;28(20):3239–47. Epub 2010/05/26. doi: 10.1200/JCO.2008.21.6457 . 20498403 10.1200/JCO.2008.21.6457

[34] Robert NJ, Dieras V, Glaspy J, Brufsky AM, Bondarenko I, Lipatov ON, et al RIBBON-1: randomized, double-blind, placebo-controlled, phase III trial of chemotherapy with or without bevacizumab for first-line treatment of human epidermal growth factor receptor 2-negative, locally recurrent or metastatic breast cancer. J Clin Oncol. 2011;29(10):1252–60. Epub 2011/03/09. doi: 10.1200/JCO.2010.28.0982 . 21383283 10.1200/JCO.2010.28.0982

[35] Gianni L, Romieu GH, Lichinitser M, Serrano SV, Mansutti M, Pivot X, et al AVEREL: a randomized phase III Trial evaluating bevacizumab in combination with docetaxel and trastuzumab as first-line therapy for HER2-positive locally recurrent/metastatic breast cancer. J Clin Oncol. 2013;31(14):1719–25. Epub 2013/04/10. doi: 10.1200/JCO.2012.44.7912 . 23569311 10.1200/JCO.2012.44.7912

[36] Cardoso F, Costa A, Senkus E, Aapro M, Andre F, Barrios CH, et al 3rd ESO-ESMO international consensus guidelines for Advanced Breast Cancer (ABC 3). Breast (Edinburgh, Scotland). 2017;31:244–59. Epub 2016/12/09. doi: 10.1016/j.breast.2016.10.001 . 27927580 10.1016/j.breast.2016.10.001

