# Supplementary figures and images for: Evaluation of drug mechanism and efficacy of a novel anti-angiogenic agent, TTAC-0001, using multi-modality bioimaging in a mouse breast cancer orthotopic model
Source: PLoS One. 2018 Jan 25;13(1):e0187063. doi: 10.1371/journal.pone.0187063 (PMC5784895; doi:10.1371/journal.pone.0187063)

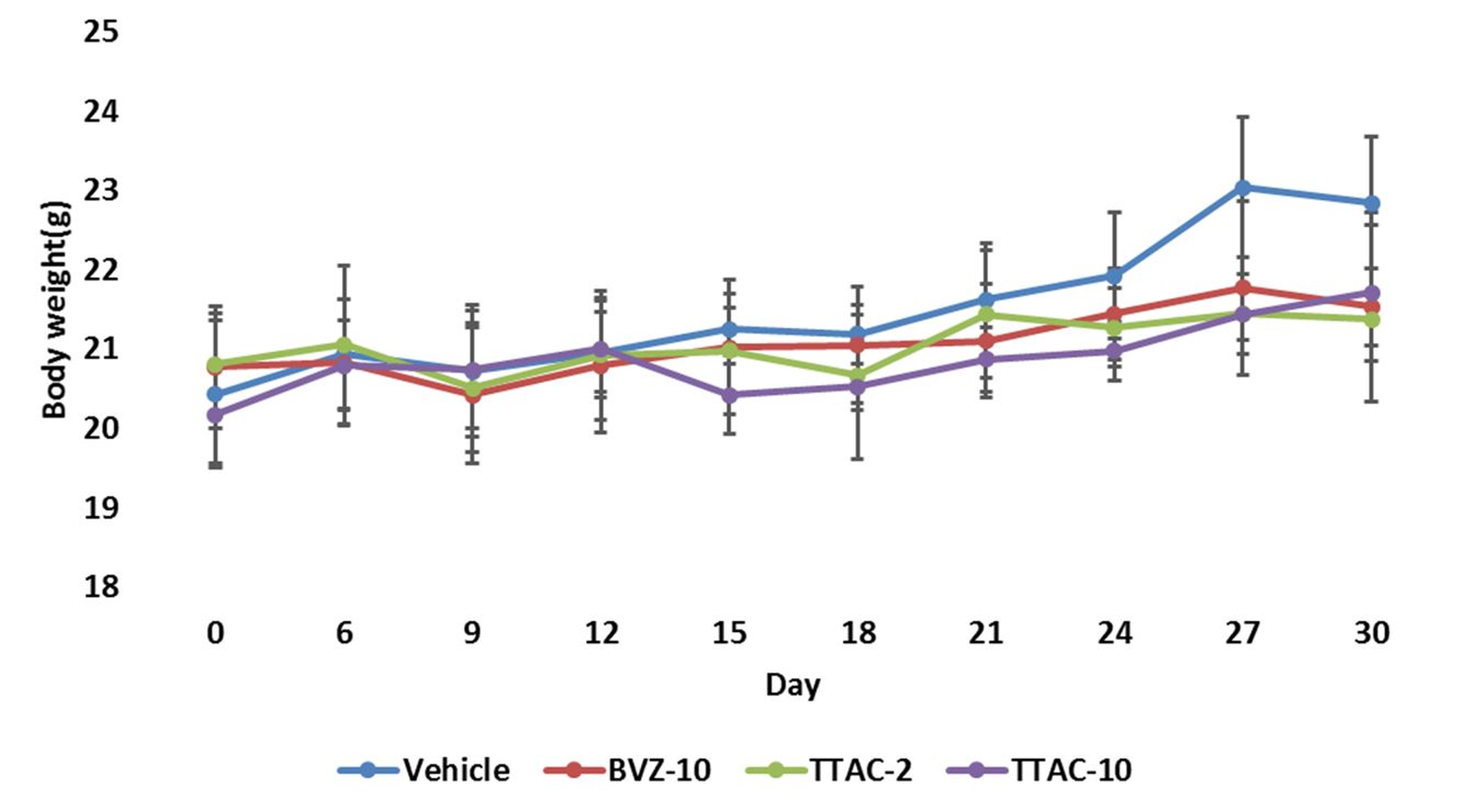

Supplement: S1 Fig — Body weight of the four groups (control, bevacizumab 10 mg/kg; TTAC-0001 2 mg/kg; TTAC-0001 10 mg/kg) was monitored prior to any treatment (0 day) and then every 3 days. (TIF) [file pone.0187063.s001.tif]

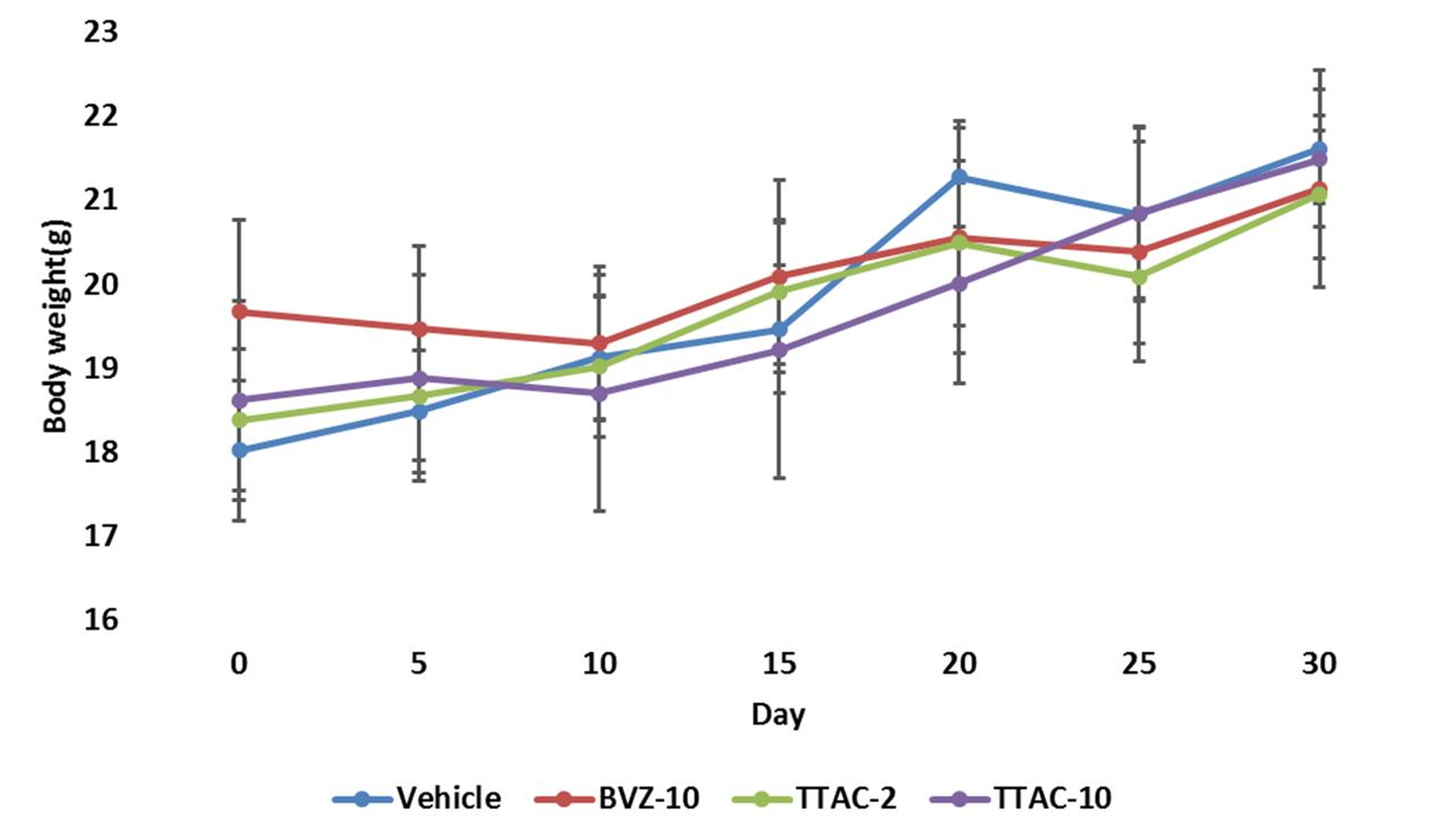

Supplement: S2 Fig — Body weight of the four groups (control, bevacizumab 10 mg/kg; TTAC-0001 2 mg/kg; TTAC-0001 10 mg/kg) was monitored prior to any treatment (0 day) and then every 5 days. (TIF) [file pone.0187063.s002.tif]
